# Stem cells and bFGF in tendon healing: Effects of lentiviral gene transfer and long-term follow-up in a rat Achilles tendon defect model

**DOI:** 10.1186/s12891-016-0999-6

**Published:** 2016-04-05

**Authors:** T. M. Kraus, F. B. Imhoff, J. Reinert, G. Wexel, A. Wolf, D. Hirsch, A. Hofmann, U. Stöckle, S. Buchmann, T. Tischer, A. B. Imhoff, S. Milz, M. Anton, S. Vogt

**Affiliations:** Department for Sports Orthopaedics, Klinikum rechts der Isar der Technischen Universität München, Munich, Germany; Institute of Molecular Immunology/Experimental Oncology and Therapy Research, Klinikum rechts der Isar der Technischen Universität München, Munich, Germany; BG Trauma Center, Eberhard Karls University Tübingen, Tübingen, Germany; Anatomische Anstalt, Ludwig Maximillians Universität, Munich, Germany; Department of Orthopaedic Surgery, University of Rostock, Rostock, Germany; Department of Sports Orthopaedics, Hessing Stiftung, Augsburg, Germany

**Keywords:** Growth factor, Mesenchymal stem cells (MSCs), Tendon healing, bFGF, Lentiviral vector

## Abstract

**Background:**

The influence of stem cells and lentiviral expression of basic fibroblastic growth factor (bFGF) on tendon healing and remodelling was investigated in an in-vivo long-term (12 weeks) rat Achilles tendon defect model.

**Methods:**

In sixty male Lewis rats, complete tendon defects (2.4 mm) were created and either left untreated (PBS) or treated by injection of stem cells lentivirally expressing the enhanced green fluorescence marker gene eGFP (MSC-LV-eGFP) or basic fibroblast growth factor bFGF (MSC-LV-bFGF). Tendons were harvested after 12 weeks and underwent biomechanical and (immuno)-histological analysis**.**

**Results:**

After 12 weeks the mean ultimate load to failure ratio (treated side to contralateral side) in biomechanical testing reached 97 % in the bFGF-group, 103 % in the eGFP-group and 112 % in the PBS-group. Also in the stiffness testing both MSC groups did not reach the results of the PBS group.

Histologically, the MSC groups did not show better results than the control group. There were clusters of ossifications found in all groups. In immunohistology, only the staining collagen-type-I was strongly increased in both MSC groups in comparison to PBS control group. However, there were no significant differences in the (immuno)-histological results between both stem cell groups.

**Conclusion:**

The biomechanical and (immuno)-histological results did not show positive effects of the MSC groups on tendon remodelling in a long-term follow-up. Interestingly, in later stages stem cells had hardly any effects on biomechanical results. This study inspires a critical and reflected use of stem cells in tendon healing.

## Background

The healing potential of tendons is reported to be inferior to bone, skin and other connective tissue [1]. In general tendon healing is relatively slower than the healing of other connective tissues and regenerated tendons are of biomechanical/histological minor quality and strength [2, 3]. However, as tendon ruptures cause a considerable loss of function effort has been made to improve primary tendon healing as well as to increase the quality of the repaired tissue. In recent research the most promising results derived from studies that applied bone-marrow-derived mesenchymal stem cells (MSC) in order to accelerate Achilles tendon healing in rabbit [4] or rat rupture models [5]. The effects of tendon healing or tendon-/bone healing with the application of MSCs, bone marrow derived cells (BMCs) or adipose tissue derived stem cells is well investigated [5–7]. MSCs may contribute to healing not only by direct differentiation but also by the release of growth factors such as IGF-1, TGF-ß, VEGF, PDGF, and bFGF [8–10]. One of the most promising aspects of stem cell application to healing tissue is furthermore the modulation of the healing process. Until today it is not exactly known when, how and in which concentration growth factors are needed to improve the healing process [2, 11].

As a another aspect, of all growth factors especially bFGF resulted in an increase of collagen-type-I and collagen-type-III production [12], which might therefore strengthen the biomechanical and histological properties of regenerative tendons. Previous short-time studies showed partially positive, partially negligible effects of MSC and bFGF after short-time investigation [13].

Therefore the aim of this study was to assess the healing potential of rat Achilles tendons in a defect model after treatment with lentiviral bFGF transduced mesenchymal stem cells (MSC-LV-bFGF) after a healing period of twelve weeks in comparison to stem cells alone. Until today the combination of mesenchymal stem cells together with viral bFGF transduction on tendon healing has only been analysed after shorter periods.

## Methods

### Study design

Sixty male Lewis-rats were divided into three groups: an eGFP-MSC group (mesenchymal stem cells lentivirally expressing enhanced green fluorescent protein), a bFGF-MSC group (mesenchymal stem cells expressing bFGF lentivirally), and a control PBS (phosphate buffered saline) group. After complete dissection of the left Achilles tendon with a 2.4 mm arthroscopy punch (Arthrex, Naples, USA) the tendon was treated randomly with injection of either PBS, MSC-LV-eGFP or MSC-LV-bFGF. The right tendon remained untreated as a control for biomechanical testing. After the healing period of 12 weeks the treated tendons were harvested. In each group, ten tendons were used for biomechanical testing, and ten underwent histological analysis.

Procedures involving animal care and treatment were conducted in conformity with the institutional guidelines that are in compliance with national and international laws (DIRECTIVE 86/609/EEC; German animal welfare law; FELASA guidelines). The ethical committee for animal experiments of upper Bavaria approved the study protocol (No. 55.2-1-54-2531-55-09).

### Stem cells

Isolation of rat mesenchymal stem cells from tibia and femur bone marrow from male Lewis rats was performed as described by Lennon et al [14]. Stem cells were cultured as described by Neuhuber et al. [15] for lentiviral transduction, differention assays and cell transplantation.

As described by Wubbenhorst et al. [16] a third generation packaging system was used to produce VSV.G-pseudotyped, self-inactivating lentiviral vectors expressing eGFP or bFGF under control of the spleen focus-forming (SF) virus promoter, respectively, by transient transfection of 293 T cells [16]. Passage 2 MSC were infected with lentiviral supernatants in presence of 8 μg/ml polybrene (Sigma-Aldrich, Germany) over night.

Characterization of rat MSC was performed according to Kraus et al. [13]. The mesenchymal stem cell characteristics had been verified by differentiation assays showing adipogenic, chondrogenic, and osteogenic differentiation potential in vitro as well as transduction efficiency and bFGF expression. bFGF transduction resulted in a sixtyfold increase of bFGF proteins in comparison with non-transduced and eGFP-transduced cells [13].

For each application a suspension of 100 μl was prepared. The minimum amount of 10^6^ MSC was assured for every injection. Suspensions were prepared at the day of surgery for immediate use.

### Animals

Fourteen week old male Lewis rats (LEW/Crl inbred) were obtained from Charles River (Sulzfeld, Germany) with a mean body weight at point of surgery of 395 ± 25 g (standard deviation). Acclimatization lasted at least 14 days. Animals were kept at room temperature of 22 ± 2 °C. Lighting was provided by a 12–hour on-off cycle. Two animals were housed together in a standard open-top Makrolon type-IV cage (Tecniplast, Italy) with autoclaved sawdust bedding and hay. Rat corner houses (Bioscape GmbH, Germany) and pulp as nesting material were offered to the rats. They had ad libitum access to water and food (Altromin, Germany). Health monitoring was performed accordingly to the recommendations of FELASA (2002) except for *Hantavirus*. In the rat unit *Pasteurella pneumotropica* and *Rat Minute Virus* were detected irregularly.

### Surgical procedures

The surgical procedures were performed under veterinary supervision. The rats were randomly dedicated to one of the three groups. Weight was measured prior to each surgery. After inducing the anaesthesia with an intramuscular injection (into the right hindlimb) of medetomidine (0.15 mg/kg), midazolam (2 mg/kg), and fentanyl (0.005 mg/kg) the left hindlimb was shaved and disinfected (Cutasept F; isopropanol; Bode, Germany). 100 % Oxygen was added to the respiratory air, a heating mat was used to prevent cooling and the vital signs were monitored. Surgery was performed under sterile conditions.

**Fig. 1:**
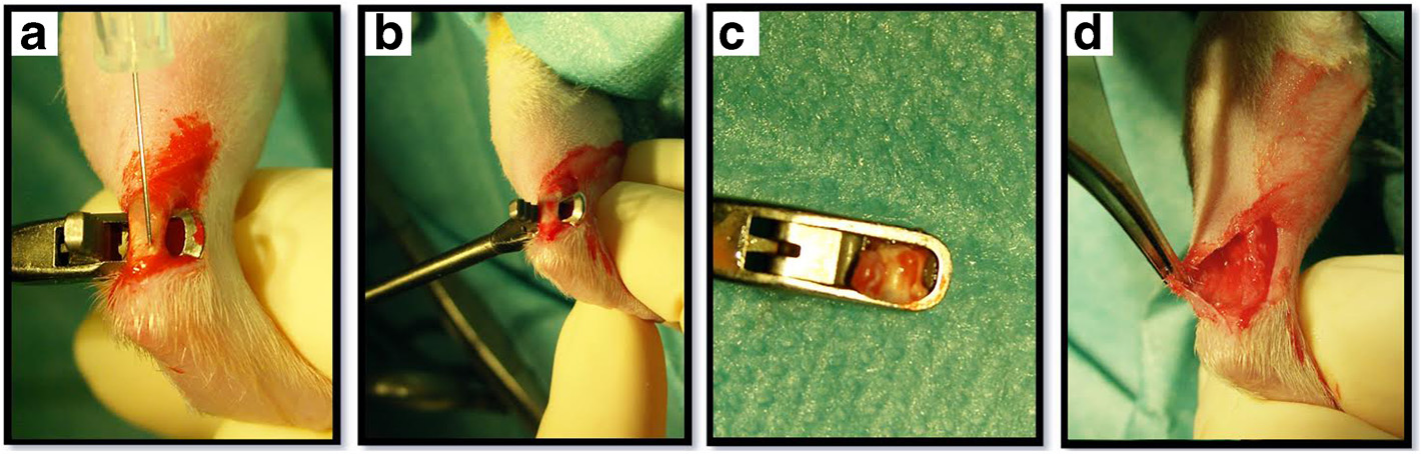
**a** injection of suspension. **b** creation of the tendon defect. **c** Punch with tendon fragment. **d** wound with tendon defect

The left Achilles tendon was laid open with a skin incision of approximately 15 mm on the medial side of the left hind limb. An arthroscopic punch (size 2.4 mm) was placed around the Achilles tendon in a distance of approximately 2 mm from the calcaneal bone (Fig. 1).

The injection of 100 μl of cell suspension proximally and distally to the designated tendon defect was performed using a syringe. Then, the tendon was completely dissected and was not sutured. The skin was sutured intracutaneously with 4-0 absorbable Monocryl (poliglecaprone; Johnson & Johnson, Germany). The suture was followed by OpSite spray-on dressing (Smith & Nephew, Germany). Afterwards animals received metamizole (200 mg/kg) s.c. for immediate postoperative analgesia.

Anaesthesia was antagonized with an s.c. injection of atipamezole (0.75 mg/kg), flumazenile (0.2 mg/kg) and naloxon (0.12 mg/kg). After awakening buprenorphine (0.05 mg/kg) was injected s.c. for analgesia. Animals received postoperative analgesia over three days after surgery (buprenorphine 0.05 mg/kg/12 h s.c. and metamizole 200 mg/kg/12 h p.o.). No immobilization was applied and no partial weight-bearing model was used throughout the healing period.

### Sample collection

After 12 weeks the rats were anesthetized with inhalation of 2 % isoflurane and then euthanized in deep anaesthesia with an intravenous overdose of pentobarbital (80 mg/kg). Both tendons were prepared and harvested as a whole with the triceps surae muscle on the proximal site and the calcaneal bone distally. Tendons for biomechanical analysis were immediately frozen (-18° Celsius). Tendons designated for immunohistological analysis were prepared carefully by removing muscle and bone. Fixation was done with 70 % methanol and tendons were stored at +8° Celsius.

### Statistical analysis

Results of the biomechanical testing after twelve weeks healing period were analyzed across treatments using one-way ANOVA. The independent variable treatment with the characteristics PBS, eGFP and bFGF defined the groups for comparison.

The results after the 12 weeks healing period were compared with the results of the previous study (14 or 28 days healing period) using two-tailed unpaired t-tests.

Statistical significance was assumed at *p* < 0,05. SPSS software was used for statistical analysis (SPSS v21.0.0.0.0 for Mac OS; SPSS Inc.).

### Biomechanical testing

**Fig. 2:**
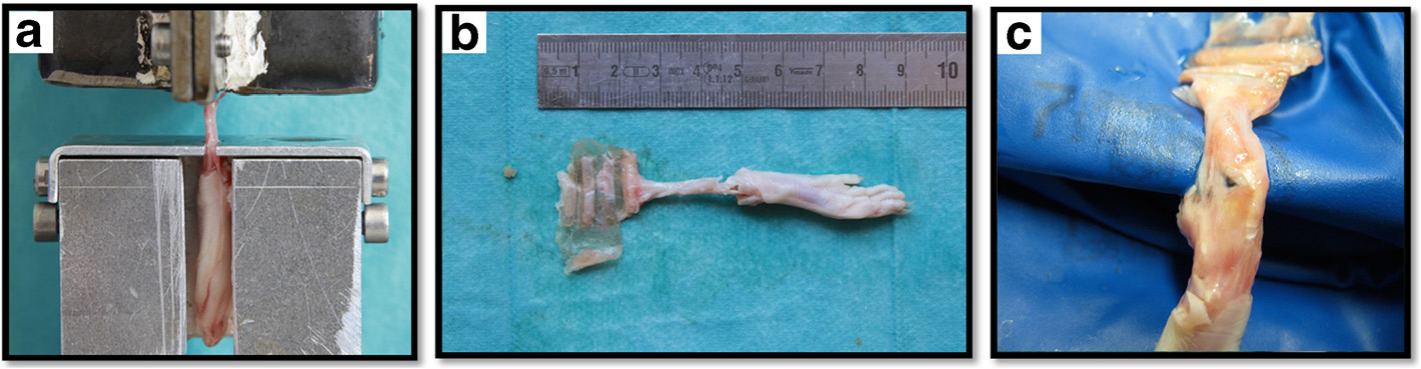
**a** specimen in testing machine. **b** macroscopic view of hind limb after testing. **c** detailed view of hind limb after testing

Thawed Achilles tendons were prepared for biomechanical testing by blunt dissection of excessive muscle tissue from the muscle-tendon-junction. The tendons underwent macroscopic assessment and standardized picture documentation. The proximal end was fixed in a cryoclamp. The distal end of the Achilles tendon was placed in a mounting grid to lock the calcaneal bone into position. For testing, a mechanical testing machine (Zwicki 1120, Zwick, Ulm, Germany) was used. Preload was set to 2 N, then the tendons were axially pulled at a constant speed of 0.16 mm/s until maximum load to failure. Room temperature was kept constant at 21° Celsius (Fig. 2).

Considering the diverse constitution of the rats, contralateral tendons were tested in the same way for percentage statistical analysis (matched pairs). SPSS software was used for calculation of stiffness and ultimate load to failure (SPSS v21.0.0.0.0 for Mac OS; SPSS Inc., IBM Company).

### Histological analysis

After methanol fixation tendons were decalcified using 10 % EDTA solution (HNaO Sodium hydroxide S5881 Sigma, Germany; EDTA A3234 AppliChem, Germany) for three to six weeks under control by X-ray (30 kV and 10 s; X-ray equipment: Cabinet x-ray systems faxitron series, HP, Germany and X-ray film: Structurix D4DW 18 × 24, NDT Systems, Agfa, Germany). Then tendons were transferred to 100 % methanol. Twenty-four hours prior to cryosectioning, tissue samples were stored in a 5 % sucrose and phosphate-buffered saline solution. Sections were sliced to thickness of twelve micrometers (Cryostat CM 1950, Leica, Germany and Cryo Gel Embedding Medium, Leica, Germany). Hematoxylin and eosin staining was performed. On the basis of a semi-quantitative four-point-scoring system (from 0 = normal to 3 = markedly abnormal appearance) in six categories: (1) fibre arrangement, (2) tendon fibre structure, (3) regional variations in cellularity, (4) increased vascularity and (5) scarring (decreased collagen staining) and (6) hyalinisation were assessed as established by Longo et al [17]. The final score results from the sum of the individual scores given in each category.

### Immunohistological analysis

Sliced tendon sections were stained using primary monoclonal antibodies for collagen-type-I and collagen-type-III (Sigma, Germany) in dilutions of 1:2000 (collagen-type-I) and 1:4000 (collagen-type-III). Evaluation of tendon staining was performed by dividing the tendons into three parts (proximal, middle and distal third). Staining grades were assessed as 0 (no staining), 1 (partial staining) and 2 (strong staining). Immunohistological analyses were performed by two blinded investigators.

## Results

### Macroscopic assessment

After 12 weeks all operated Achilles tendons were completely recovered. No infections or other complications occurred. Macroscopic assessment showed sporadic indurations in all groups within the former defect zone.

### Biomechanical testing

#### Ultimate load to failure

**Fig. 3:**
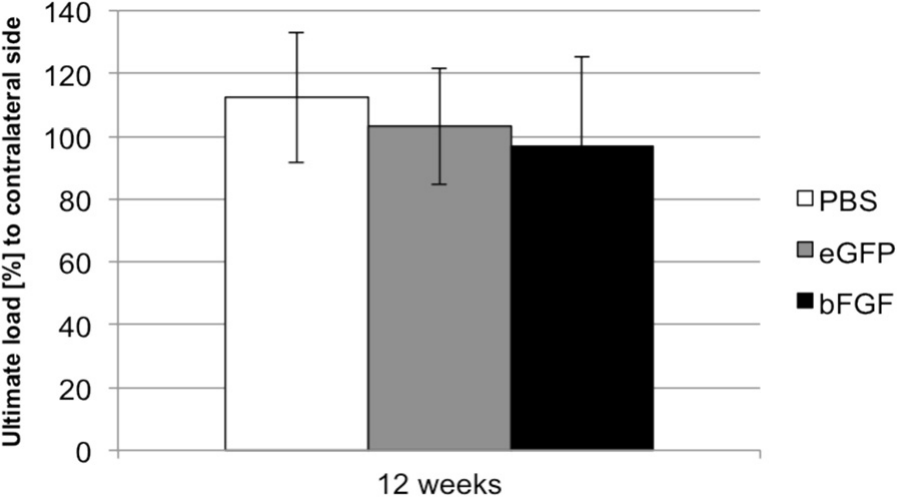
Ultimate load [%] to contralateral side (mean + standard deviation)

The mean ultimate load to failure ratio (treated side to contralateral side) reached 97 % in the bFGF-group, 103 % in the eGFP-group and 112 % in the PBS-group after the 12 weeks healing period (Fig. 3). However, the differences between all groups were not significant (*p* = 0.310 for PBS versus eGFP; *p* = 0.184 for PBS versus bFGF; and *p* = 0.567 for eGFP versus bFGF).

#### Stiffness

**Fig. 4:**
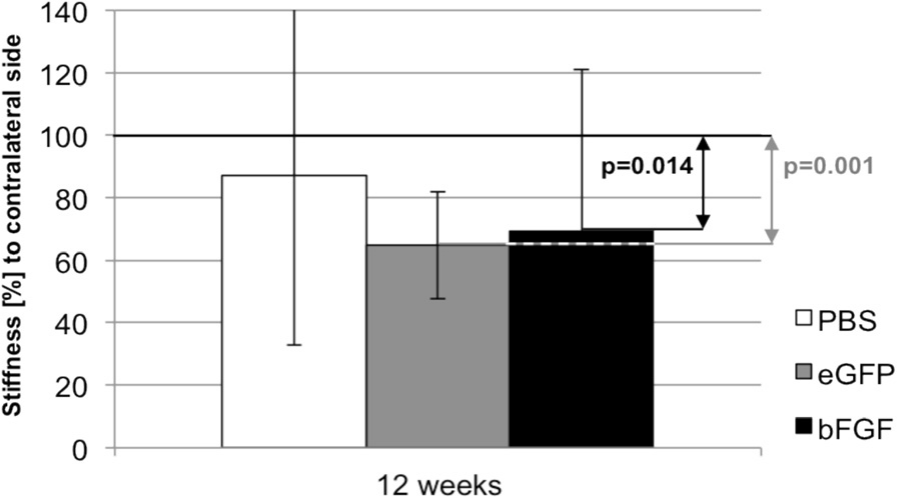
Stiffness [%] to contralateral side (mean + standard deviation)

Stiffness of the operated tendons in comparison to the contralateral untreated tendons (stiffness ratio) was significantly lower across all groups (*p* = 0.000), in the MSC-LV-eGFP-group (*p* = 0.001) and the MSC-LV-bFGF-group (*p* = 0.014). A trend was observed in the PBS-group but the difference was not significant (Fig. 4; *p* = 0.070). Statistical analysis after the 84 days healing period did not show any significant differences between all groups.

### Histological analysis

**Fig. 5:**
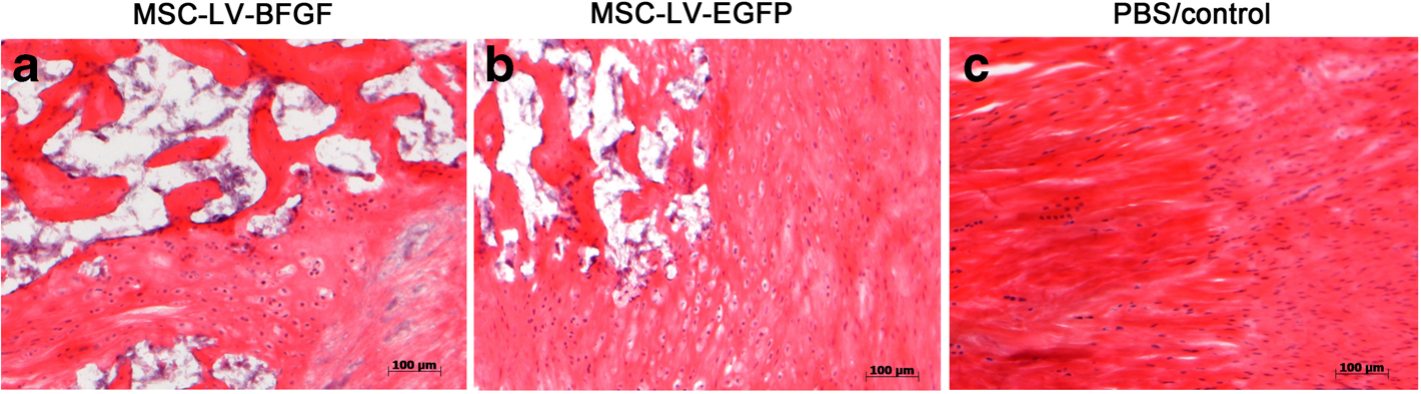
Hematoxylin and eosin staining of the tendons after healing period of twelve weeks. Fibre structure remodelling is shown with chondral ossifications. The MSC pictures show large amounts of ossifications (white clusters). These white clusters do not show a specific variation or spreading in **a**, **b** or **c**

Assessment of hematoxylin and eosin staining showed no significant differences between groups (Fig. 5 and Table 1). Almost all tendons showed chondral ossification. Dispositions of ossifications varied between separate, clustered and bar-shaped foci in the whole tendon structure. Consistent distribution patterns could not be identified. Tendon fibre structures were altered substantially by ossifications. The semi-quantitative scoring does not show relevant differences in the groups, also not in the specific sub-categories.

**Table 1:** Haematoxilyn and eosin staining after 12 weeks. (*p*-value n.s.)

Group	Number of tendons		Score median
	Valid	Missing	
bFGF	10	0	10 (7–10)
eGFP	10	0	10 (8–11)
PBS	10	0	9.5 (5–10)

Semi-quantitative scoring in six categories (0–18 points overall; 0 = normal – 3 = abnormal per category)

**Fig. 6:**
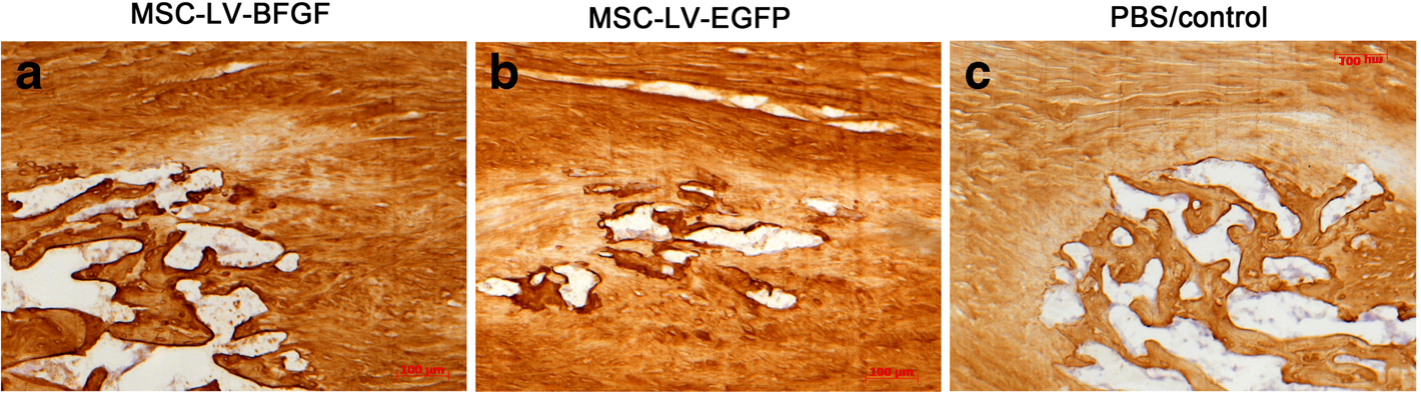
Collagen-type-III staining of tendons after the healing period of 12 weeks. Ossifications (white clusters) after remodelling in all groups (**a**, **b**, **c**)

In the immunohistological analysis collagen-type-I and collagen-type-III staining showed no significant differences between groups (Fig. 6). Foci of ossification mostly appeared positive for collagen-type-I but negative for collagen-type-III. Remaining parts of tendons stained strongly for collagen-type-I and collagen-type-III.

## Discussion

The most important finding of this study was that in all study groups biomechanical testing showed very good healing potential achieving normal tendon strength regarding load to failure values compared to the contralateral tendon. However, all treated tendons gained inferior results in stiffness. Lentiviral bFGF expression in mesenchymal stem cells (MSC-LV-bFGF) did not significantly affect the biomechanical or histological and immunohistological results in comparison to MSC-LV-eGFP group or the control group in this long-term rat Achilles tendon model.

The histological analysis presented no improvement of fibre structure after a long-term remodelling period. Interestingly clusters of ossification were discovered in almost all specimens throughout the study groups.

In literature one of the most promising approaches to accelerate healing is the application of mesenchymal stem cells [4–6, 18]. Besides their proliferation and differentiation capacity into osteoblasts, chondrocytes, adipocytes and fibroblasts [8] the beneficial effect of MSCs may not be the differentiation into the regenerating tissue type, but rather the secretion at sites of injury of growth factors such as IGF-1, TGF-ß, VEGF, PDGF, and bFGF [9, 19, 20]. Therefore, not only the differentiation potential but also the release of growth factors distinguishes MSCs from tenocytes or fibroblasts. In this context positive effects in the early stage of tendon healing were found by Chan et al. [12]. Their study showed a positive effect on histological and immunohistological repair characteristics only after seven days, using bFGF as a one- time administered protein for patellar tendon healing in a rat model.

In another recently published study in a rat rotator cuff model adipose derived stem cells did not improve the healing potential neither in the histological assessment nor in the biomechanical investigation [7].

In a previous study [13] partially positive effects were detected when MSC and bFGF expressed significantly high values of collagen-type-I after two or four weeks. However, although there were high amounts of collagen-type-I in the MSC groups in short term assessment long-term remodelling did not show significant differences in tendon healing in this rat model. However, the ultimate load to failure ratio showed a significant improvement in the PBS-group from day 14 to day 84 (*p* = 0.000) and in the eGFP-group a significant increase from day 14 to 84 (*p* = 0.004) [13]. The ultimate load to failure ratio showed also improved results in the bFGF-group, however, these findings were not significant (*p* = 0.051 from day 14 to 84).

Ossification occurs as a known process in tendon remodelling [3, 11]. Zhang et al. [21] showed in a tenotomy rat achilles tendon model investigating the effect of a selective cox2 inhibitor that heterotopic ossification developed in 100 % of the non treated group after 10 weeks. Similar findings are described by Pietschmann et al. [22] in a tendon defect model with the use of polyglycol acid and collagen-type-I scaffolds, seeded with mesenchymal stem cells or tenocytes. Central ossification and tendon-like tissue were observed after 16 weeks in the superficial tendon layers in all study groups. Chondral metaplasia and endochondral ossification also occurred in the healed tissue of both groups at 60 and 90 days in an Achilles tendon rat model with tenotomy, suture and application of a gel with elastin-derived peptide in the treated group [23]. Already in 1969, Salah et al. [24] described heterotopic ossification in the Achilles tendon of the rat following crushing and ligation. In a recently published study by Lui et al. ectopic ossifications were not seen after transplantation of allogeneic tendon-derived stem cells (TDSC) in rat patellar tendon model. However, biomechanical outcome is not reported [25]. As a possible explanation for the suppression of ossifications weak immunoreactions and anti-inflammatory effects of the TDSCs are reported [26]. As recently reported the implantation of a surgical mesh loaded with MSCs might be a promising approach. Schon et al. found better histological results in a rat Achilles tendon model in the mesh/MSC group than in the mesh group or the control group alone. However, a biomechanical investigation was not performed [27]. New research has been performed with a tendon-derived, extracellular matrix hydrogel in order to direct tendon regeneration also in a rat Achilles tendon defect model [28]. In biomechanical assessment in comparison to the contralateral side, treated with saline, there were differences between the groups observed after four weeks; however no differences after two or eight weeks. In summary application of stem cells and biologicals seems to have positive effect in mid-term investigation at time points between two to four weeks, but no longer lasting effects in rat Achilles tendon models. A further promising approach might be the application of calcium alginate gels as stem cell matrix. Schmitt et al. have observed paracrine stem cell activity and quantified concentrations of bFGF and VEGF in cell culture supernatants. The gels may function as immobilization matrices for MSCs and therefore enhance healing after surgery [29].

In summary, no general conclusions about biologicals in tendon healing can be drawn as tissue from individual to individual differs and the healing potential of a young, healthy rat might also be different than in a clinical setting with degenerative tendons in humans [30]. However, this study is the first to report the outcome after a long healing period of 12 weeks in a rat tendon model. An investigation over such a long period has so far not been performed, but still further investigation seems necessary to finally develop promising approaches.

## Conclusion

The biomechanical and (immuno)-histological results did not show positive effects of the MSC groups on tendon remodelling in a long-term follow-up. Interestingly, in later stages stem cells had potentially negative effects on biomechanical results in comparison to two or four week results. Chondral ossification occurred in all groups after a twelve-week healing period.

### Data availiability statement

The datasets supporting the conclusions of this article are included within the article in Figs. 3 and 4 and Table 1.

## Abbreviations

bFGF: basic fibroblastic growth factor
BMC: bone marrow derived stem cells
IGF-1: insulin like growth factor
LV: lenti-viral/lenti-virus
MSC: mesenchymal stem cells
MSC-LV-eGFP: mesenchymal stem cells lentivirally transduced expressing enhanced green fluorescent protein
MSV-LV-bFGF: mesenchymal stem cells lentivirally transduced expressing bFGF
PBS: phosphate buffered saline
PDGF: platelate derived growth factor
TDSC: tendon-derived stem cells
TGF-ß: transforming growth factor beta
VEGF: vascular endothelial growth factor
VSV: vesicular stomatitis virus
